# The use of combined cognitive training and non-invasive brain stimulation to modulate impulsivity in adult populations: a systematic review and meta-analysis of existing studies

**DOI:** 10.3389/fpsyt.2024.1510295

**Published:** 2024-12-09

**Authors:** Najat R. Khalifa, Yousef Alabdulhadi, Pilar Vazquez, Charlotte Wun, Peng Zhang

**Affiliations:** ^1^ Department of Psychiatry, Queen’s University, Kingston, ON, Canada; ^2^ Department of Public Health Sciences, Queen’s University, Kingston, ON, Canada

**Keywords:** cognitive training, non-invasive brain stimulation, impulsivity, transcranial direct current stimulation, transcranial magnetic stimulation

## Abstract

**Introduction:**

Impulsivity, a tendency to act rashly and without forethought, is a core feature of many mental disorders that has been implicated in suicidality and offending behaviours. While research supports the use of non-invasive brain stimulation (NIBS) techniques, such as transcranial direct current stimulation (tDCS), to modulate brain functions, no studies specifically reviewed the use of combined cognitive training and NIBS to modulate impulsivity.

**Methods:**

We aimed to conduct a systematic review and meta-analysis to synthesise the literature on the use of combined cognitive training and NIBS to modulate impulsivity and its subdomains (motor, delay discounting, reflection). We searched Scopus, PsychInfo, Medline, and Cinahl electronic databases, dissertations database, and Google scholar up to September 2024.

**Results:**

Following the Preferred Reporting Items for Systematic Reviews and Meta-Analyses (PRISMA) guidelines, four randomised controlled studies involving the use of combined cognitive training and tDCS in 127 subjects were included in the study. These studies included subjects with substance use disorders, obesity, and Parkinson’s disease. Meta-analysis showed that combined cognitive training and tDCS had no statistically significant effects on motor impulsivity as measured using reaction times on the Stop Signal Task and Go/No Go tasks. One study that measured impulsiveness scores on a delay discounting task also showed no significant results. No studies measured reflection or cognitive impulsivity.

**Discussion:**

There is a dearth of literature on the use of combined cognitive training and NIBS for impulsivity. This in conjunction of clinical heterogeneity across studies makes it difficult to draw definitive conclusions about the neuromodulation of impulsivity and its subdomains using combined cognitive training and NIBS. The findings of this study highlight the need to conduct more studies in the field.

**Systematic review registration:**

https://www.crd.york.ac.uk/prospero/, identifier CRD 42024511576.

## Introduction

### Overview

Impulsivity is a multifaceted construct that reflects a tendency to act rashly and without forethought, with negative consequences for the individual and others (1, 2). Trait impulsivity refers to personality traits and behaviors encompassing urgency, sensation seeking, lack of perseverance, and disinhibition (3). Behavioral manifestations of impulsivity are multifaceted, including deficits in response inhibition (motor), delay discounting (temporal), and information sampling (reflection or cognitive). Additionally, Impulsivity overlaps significantly with decision making (4).

Several neuronal circuits in the brain have been implicated in impulse control. Motor impulsivity is regulated by a fronto-subcortical circuit involving the right inferior frontal gyrus, the anterior cingulated cortex, the basal ganglia, and the presupplementary motor area (5, 6). The ventromedial prefrontal cortex has been implicated in cognitive impulsivity and decision making under conditions of risk or ambiguity (7, 8). In contrast, temporal impulsivity is regulated by a fronto-limbic circuit including the anterior cingulated cortex, the ventromedial prefrontal cortex, and the nucleus accumbens of the ventral striatum (9). Dysfunction in the thalamo-cortico-striatal neural network has also been implicated in impulsivity (10).

Trait impulsivity is measured using questionnaires such as the Barrett Impulsiveness Scale [BIS-11; (11)] and the Urgency-Premeditation-Perseverance-Sensation Seeking-Positive Urgency (UPPS-P) impulsive behavior scale (3), whereas state impulsivity is measured using behavioral measures such as the Stop Signal Task (12), Delay Discounting Task (13), Go-No-Go tasks (14), and Information Sampling Task (15).

Furthermore, impulsivity is a key feature of many of the externalizing disorders listed in the fifth edition of the Diagnostic and Statistical Manual of Mental Disorders [DSM-5; (16)] including attention deficit hyperactivity disorder, antisocial personality disorder, borderline personality disorder, substance use disorder, and pathological gambling. It is also an important consideration in risk assessment tools owing to its association with aggression and suicidality (17). Impulsivity along with craving, and neurocognitive deficits have also been implicated in maintaining addictive behaviors in people with substance use disorder (18, 19). Additionally, impulsivity has been associated with poor treatment adherence in people with cocaine use disorder (20).

### Interventions to reduce impulsivity

A range of interventions have been developed to reduce impulsivity in the context of addiction including computerized cognitive training, cognitive remediation, pharmacological interventions (e.g., modafinil, galantamine), and mindfulness relapse prevention (21, 22) with some promising results. Cognitive control training has been also showed to reduce emotion-related impulsivity in adults (23). Additionally, a range of pharmacological interventions are used in clinical practice to reduce impulsivity, with some evidence supporting the use of fluoxetine, carbamazepine, and topiramate for impulse control disorders (24) and quetiapine for impulsivity in people with borderline personality disorders (25).

### NIBS

Non-invasive brain stimulation techniques (henceforth referred to as NIBS) include transcranial direct current stimulation (tDCS), transcranial random noise stimulation (tRNS), transcranial alternating current stimulation (tACS), transcranial magnetic stimulation (TMS), deep brain stimulation (DBS), and others. While tRNS and tACS are primarily used as investigative tools in research, tDCS and TMS have been used for the treatment of various neuropsychiatric disorders such as depression, obsessive compulsive disorder, substance use disorder, schizophrenia, and Parkinson’s disease (PD) amongst others (26–28). Additionally, data support the use of tDCS to improve substance use-related outcomes by reducing craving and relapse rates (29), highlighting its potential as a non-pharmacological option for substance use disorders (30). Moreover, the utility of NIBS techniques in reducing impulsivity has been demonstrated in systematic reviews in both healthy subjects (31) and people with mental disorder (32), although the results are often inconsistent across studies. While the exact mechanism of action of NIBS is not fully understood, their effects are thought to be related to inducing neuroplasticity changes in the brain (33) and modulating the function of various neurotransmitters (34).

### Cognitive training

According to Gobet and colleagues (35), “Cognitive training refers to interventions using cognitive tasks or intellectually demanding activities, the goal of which is to enhance general cognitive ability.” Cognitive training encompasses a wide range of activities including performing cognitive tasks, learning music, and playing videogames (35). Central to cognitive training are the concepts of near transfer (i.e., the generalization of acquired skills across two or more related domains) and far transfer (i.e., the generalization of acquired skills across loosely related domains) (35, 36).

The utility of cognitive training in promoting neuroplasticity changes in the brain has been demonstrated in addiction disorders (37). Cognitive training has been shown to have a modest effect on enhancing cognitive functioning in people with mild to moderate PD especially in areas of memory, processing speed, and executive functions (38). Cognitive rehabilitation has been shown to improve cognitive performance and resting functional brain connectivity and reduce functional disability in PD (39). Despite growing interest in cognitive training and a multi-billion-dollar industry, empirical evidence in the field remains inconclusive (35, 36).

### Current study

There is some evidence to support the use of cognitive training to enhance the effects of tDCS both on trained tasks and non-trained but related tasks (30, 40). Indeed, the neuroplasticity-related effects of tDCS are enhanced when tDCS is administered over an already engaged brain region while engaged in a cognitive training task (41). For example, a recent review reported small but statistically significant effects for combined cognitive training and tDCS on attention, working memory, language and global cognitions in people with neuropsychiatric disorder (42). Although previous reviews showed positive effects on cognitive functioning for combined Transcranial Electrical Stimulation (tES) and cognitive training (43) as well as combined cognitive training and tDCS (42), it is unclear if the putative advantage of combing cognitive training and NIBS will hold true for impulsivity. This is important since the existing evidence suggests that combined cognitive training and tDCS, for example, can have a synergistic positive effect on behavior (44–46).

Given the potential negative consequences of impulsivity (1) and the absence of published reviews that specifically examined the effects of combined NIBS and cognitive training on impulsivity, we aimed to conduct a systematic review and meta-analysis to fill this gap in the literature. Conducting a review of this kind would help not only to enhance knowledge, but also to identify areas for future research with a view to developing adjunctive interventions for impulsivity. Although a range of pharmacological and psychosocial interventions have been developed to target impulsivity in various disorders, these are not without limitations. Take attention deficit hyperactivity disorder as an example. A longitudinal study of ten European countries showed that while medications showed positive effects on attention deficit hyperactivity disorder symptoms, the effects of psychosocial interventions were either insignificant or negative (47). Although more recent studies highlighted the beneficial effects of psychosocial interventions (48) and demonstrated relatively large effect sizes in the short-term in populations with attention deficit hyperactivity disorder, there is still a need to enhance current therapeutic strategies for this patient population (49). Put together, these findings emphasize the need to develop adjunctive interventions, such as combined cognitive training and NIBS, for disorders that are marked by impulsivity.

## Methods

### Protocol and registration

The review protocol is enrolled in the PROSPERO international prospective register of systematic reviews (CRD42024511576) and reporting follows the Preferred Reporting Items for Systematic Reviews and Meta-Analyses (PRISMA) (50).

### Inclusion criteria

The PICO framework was used to determine inclusion criteria. Studies were included in the review if they (i) involved adult participants with or without mental disorder; (ii) involved the use of combined cognitive training and active NIBS (e.g., tDCS, TMS, DBS, tES); (iii) included a control group such as combined cognitive training and sham NIBS; and (iv) used at least one behavioral tool to measure overall impulsivity or its facets and reported the findings of these tools. We included studies involving participants with mental disorder since impulsivity is a core feature of some of the mental disorders classified in the Diagnostic and Statistical Manual of Mental Disorders fifth edition (DSM-5) including attention deficit hyperactivity disorder, borderline personality disorder, and antisocial personality disorder (16). Studies involving children and adolescents, editorials, non-controlled studies, and those in languages other than English were excluded. No other specific exclusion criteria were applied for setting, region, or date of publication.

### Search strategy

The initial search covered the period August 1980 until September 2024 and included Scopus, PsychInfo, Medline, and CINHAL electronic databases in addition to dissertations databases (ProQuest Dissertations and Theses Global, Open Access Theses and Dissertations) and Google Scholar. The lists of references of the included studies were hand searched to identify studies not picked up by the initial search. Grey literature was also explored using Google.

We defined cognitive training in accordance with previous studies [e.g., (35, 42, 51)], including cognitive tasks designed to enhance specific domains such as working memory, decision making, attention, executive function, and others. We conceptualized impulsivity and its subdomains (e.g., motor, temporal, and cognitive or reflection impulsivity) in accordance with previous literature [e.g., (4, 52, 53)]. Appropriate behavioral measures were identified to measure motor impulsivity (e.g., Stop Signal Task, Go-No-Go task, and the Stroop Color and Word Test), temporal impulsivity (e.g., Delay Discounting Task), and reflection or cognitive impulsivity (e.g., Information Sampling Task).

### Search terms

The search terms were adapted from previous studies (4; e.g., 42) and included, “Transcranial magnetic stimulation”, “TMS”, “theta burst stimulation”, “TBS”, transcranial Direct Current Stimulation”, “tDCS”, “transcranial Electrical Stimulation”, “tES”, Random Noise Stimulation”. “RNS”, “transcranial Alternate Current Stimulation”, and “tACS”, COMBINED with “cognitive or memory” adjacent to (train*” or remediate* or enhancement* or rehabilitate* or treatment* or therapy*) AND “impulsive*”, “self-regulation”, “inhibitory control”, “impulse control”, “delay discounting”, “response inhibition”, “information sampling”, “stop signal”, “temporal discounting”, “stroop”, “go-no-go”.

### Study selection

Using the Covidence workflow platform (http://www.covidence.org), three reviewers independently screened titles and abstracts to identify potentially eligible articles. Disagreements were resolved by consensus. Full texts of potentially eligible articles were reviewed to identity a final list of studies for inclusion.

### Data extraction

Data extraction was conducted using a data collection tool designed for this study which gathered information about study title, publication date, country of origin, setting, study design, participant characteristics (gender, age), interventions, and outcome measures as well as study limitations.

### Synthesis of results

Data were pooled using random-effects meta-analysis in the Statistical Package for Social Studies (SPSS) version 27. Random-effects meta-analysis models assume that treatment effects vary across studies due to differences between the studies in addition to sampling variability. This is appropriate due to the high degree of clinical heterogeneity across the studies included in this review (54).

Evidence suggests that impulsivity is heterogenous, and that motor-, temporal-, and reflection-impulsivity should be considered separately, since the different domains reflect different types of behaviors and thus different underlying processes (55). Since subdomains of impulsivity have distinct neurobiological underpinnings and owing to differences in the mechanism of action of NIBS techniques, we aimed to conduct separate metanalyses by domains of impulsivity and type of NIBS technique to minimize heterogeneity where possible.

The principal effect size was represented as Hodge’s (adjusted) g and 95% confidence intervals (CI) by calculating the mean differences between experimental (combined cognitive training and active NIBS) and control (combined cognitive training and sham NIBS sham) conditions in post-stimulation evaluations divided by the pooled standard deviation (a summary measure known as the standardized mean difference) multiplied by a correction factor for small samples. Effect sizes were considered negative if the active intervention was in the predicted direction, and positive otherwise. If a study used multiple NIBS stimulation sites, each simulation site trial was used as the unit of analysis in meta-analysis. To minimize the risk of heterogeneity, reaction time was used if studies provided multiple outcomes (e.g., reaction time, subscale scores, performance, or accuracy). Additionally, only data relating to the immediate post-stimulation time point were included in the analysis in cases where outcome measures were assessed at different time points. Only one study in this review measured outcomes at more than one post-stimulation time point (56). For studies involving more than one control condition, only data pertaining to combined active NIBS and cognitive training and combined sham NIBS and cognitive training were included in the analysis.

### Assessment of heterogeneity

**Figure 1:**
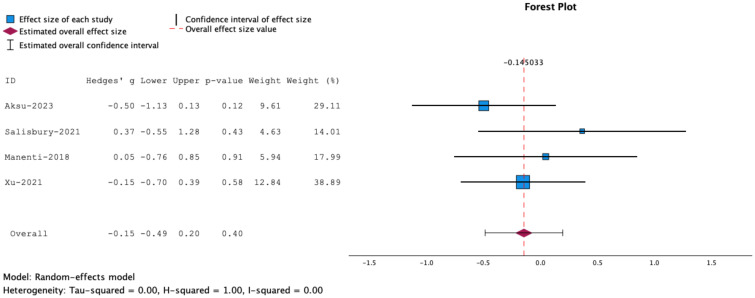
Forrest plot representing meta-analysis of inhibitory control tasks. Measures of inhibitory control: Stop Signal Task, Go/No Go and Flanker Task. The overall effect size is not statistically significant.

Between-study heterogeneity was assessed using the *H^2^*, Q, *I^2^*, and *tau^2^* statistics (57). The *I^2^* statistic reflects the percentage of variation related to heterogeneity rather than chance. The *Q* statistic represents the weighted sum of squared differences between individual study effects and the pooled effect across studies, while *H^2^* represents the relative excess in Q over its degree of freedom (58). In contrast, the *tau^2^* statistic is represents the variance of the true effect sizes (59). Heterogeneity values are described in the Forest plot (**Figure 1**). For this review, *I^2^* values of <25% were considered ‘low’; *I^2^* values >40% and *p* values of ≤0.05 were considered ‘moderate,’ and *I^2^* values >75% and *p* values of ≤0.05 were considered ‘high’.

Funnel plots were used to test for the presence of publication bias. Where publication bias was detected, the Trim and Fill procedure was applied to examine study clustering and attempt to correct for this (60) while acknowledging that a symmetrical funnel plot and non-significant tests for funnel plot asymmetry do not necessarily exclude the possibility of publication bias.

### Quality assessment

Quality assessment was conducted using the NIH National Heart, Lung and Blood Institute’s Quality Assessment Tool for Controlled Intervention Studies (61). Each study was assessed against predefined criteria that covered the domains of power calculation, randomization, allocation concealment, blinding, adherence to interventions, dropout rates, intention-to-treat analysis and others. Each study was assigned an overall quality rating (good, fair or poor) based on the total number of criteria that each study met and the potential impact of not meeting these criteria on the results.

## Results

### Search results

**Figure 2:**
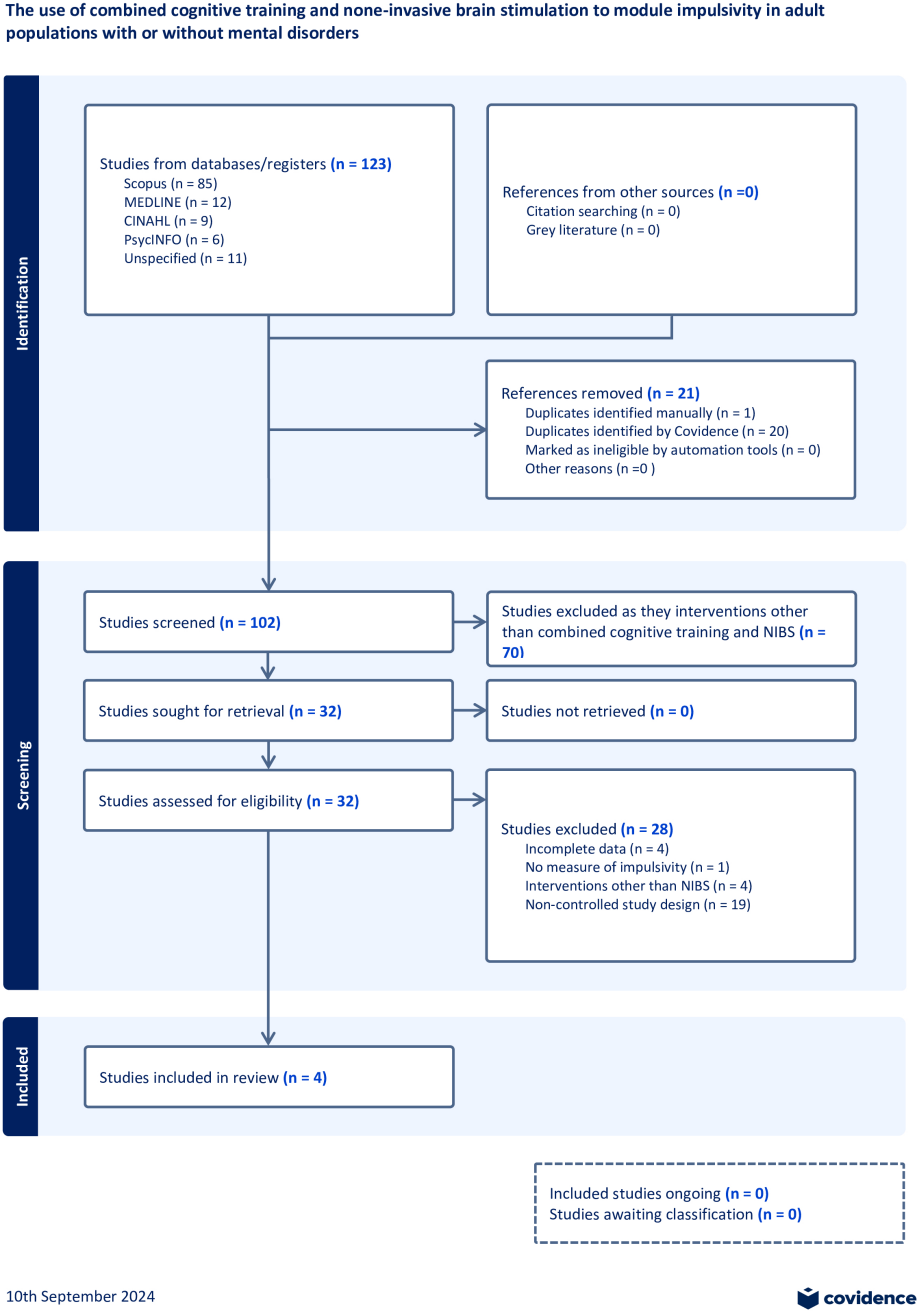
PRISMA flow diagram.

The initial search identified 123 records. After removing duplicates, 102 records remained for screening of which only 4 studies were included in the review. The PRISMA flow diagram provides more information about the rationale for exclusion (**Figure 2**).

### Study characteristics

**Table 1** summarizes study characteristics. Four randomized controlled studies involving the use of combined cognitive training and tDCS and 127 subjects were included in the study. Of those, two studies examined the efficacy of combined cognitive training and tDCS for substance use disorders such as opioid use disorder (62) and methamphetamine use disorder (56), one for Parkinson’s disease (63), and one for obesity (64). No none of these studies enrolled participants primarily based on impulsivity or used impulsivity as a primary outcome measure. All four studies targeted the dorsolateral prefrontal cortex (DLPFC) for tDCS stimulation. A range of cognitive training tasks were used including the Game of Dice Task, Brain HQ Computerized Cognitive Training, and computerized cognitive addiction therapy. Impulsivity was measured using various behavioral tasks such as the Stop Signal Task, Go/No Go task, Dot Counting Task, and Dot Probe Task. A wide range of other tools were used to measure cognitive functioning, mental health symptoms, global functioning, and quality of life.

**Table 1:** Study characteristics.

Author (year) Country	Study design	Sample and setting	Age Mean (SD)	Gender (M:F:other)	Active intervention Number of participants	Stimulation protocol (stimulation site, current intensity, duration, number of sessions)	Sham method Number of participants	Tasks and outcome measures	Limitations
Aksu (62) Turkey	Triple-blind RCT	Patients with Opioid Use Disorder recruited from a Buprenorphine Naloxane Maintenance Therapy program	30.00 (12.5)	36:2	Anodal tDCS Plus Game of Dice Task (GDT) administered 3 times during tDCS session n = 19	Right anodal and left cathodal over the DLPFC, 2 mA, 20 min, 1 session	tDCS plus GDT tDCS applied for 30 seconds at the beginning and 30 seconds at the end of the stimulation period n = 19	SST (reaction time) BART task IGT Stroop Test Digit Span (forward and backward) LNS subtests of WAIS Phonemic, Semantic, and alternating fluency tests TMT A & B The Penn Drug Craving Scale BDI BAS	Small sample, no power calculation, mainly males, population with OUD.
Manenti et al. (63) Italy	Double-blind RCT	Parkinson’s Disease patients	Sham: 63.8 (7.1) Active: 65.5 (6.4)	12:10	Anodal tDCS plus Computerised Cognitive Training (CCT) – Brain HQ N = 11	Left DLPFC, 2 mA, 25 min, 10 sessions over 2 weeks	Sham tDCS plus CCT tDCS applied for 10 s in the beginning and 10 s at the end of the stimulation period N = 11	Go/NoGo (correct responses and reaction time) Global cognitive abilities – MMP and PD-CRS Memory-Rey Auditory Verbal Learning Test, immediate and delayed recall, Digit Span Forward and Backward. Language - Phonemic and semantic verbal fluency, objects and actions picture naming of IPNP Attention and executive functions -TMT of Attentional Performance, Stroop Test, and FAB. Clinical evaluation - BDI-II, PDQ-39, Apathy Evaluation Scale, RBDSQ, UPDRS-III. and Hoehn & Yahr Scale.	Under powered due to small sample, PD population, baseline intergroup differences in cognition.
Salisbury (64) USA	Pilot Study: Randomised Controlled Trial	Adults with obesity	Active intervention: 56.5 (13.22) Sham group: 58.78 (12.44)	8:3:0	Left cathodal and right anodal tDCS BrainHQ (attention, processing speed, working memory) N= 8	DLPFC, 2mA, 2x 13 min sessions with 20 min simulation break in between 10 total sessions over 5 consecutive days	Identical to the treatment group Current only flowed the initial 30 seconds N=9	BES MN-BEST PHQ NIH Examiner: Working memory-DCT (correct responses) Impulsivity-The Flanker test (reaction time, correct responses) Cognitive flexibility-Set Shifting (correct responses) Executive task-The Unstructured Task (puzzle completion)	Small sample, baseline differences in characteristics, population validity (only adults with obesity) no power calculation
Xu et al. (56) China	Randomised Controlled Trial	Female adults with methamphetamine use disorder	Active intervention: 34.33 (6.91) Sham group: 33.5 (5.65)	0:1:0	Right Anodal & left Cathodal tDCS plus Computerized Cognitive Addiction Therapy (CCAT) N=24	DLPFC, 1.5 mA, 20 min, 10 sessions	Sham tDCS plus CCAT. tDCS current ramped up to 1.5mA in 15s and then immediately ramped down in 15s n=26	Craving: VR-based cue-induced craving measure Cognitive functions: Attention bias-Dot Probe Task (reaction time) Impulsivity-Stop Signal Task (reaction time) Delay-discounting Task (delay time) CogStat Battery: ISLT (correct responses) TWOB (accuracy)	Small sample, no power calculation, female only sample

BART, Balloon Analogue Risk Task; BAS, Beck Anxiety Scale; BDI, Beck Depression Inventory; BES, Binge Eating Scale; Brain HQ, An interactive computer software package where participants work through a series of structured exercises designed to stimulate neuroplasticity; BIS-11, Barratt Impulsiveness Scale; DCT, Dot Counting Test; DLPFC, Dorsolateral prefrontal cortex; FAB, Frontal Assessment Battery; IGT, Iowa Gambling Task; ISLT, International Shopping List Task; IPNP, International Picture; MMP, Mini Mental Parkinson’s; MN-BEST, Minnesota Blast Exposure Screening; NIH, National Institute of Health; NP, Naming Project; LNS, Letter Number Sequencing; OUD, Opioid Use Disorder; PDQ, Patient Health Questionnaire; PD-CRS, Parkinson’s disease-cognitive rating scale; PDQ-39, Parkinson’s Disease Quality of Life Questionnaire-39; RBDSQ, REM Sleep Behavior Disorders Screening Questionnaire; SST, Stop Signal Task; tDCS, transcranial Direct Current Stimulation; TMT, Trail Making Test; TWOB, Two Back Task; UPDRS-III, Unified Parkinson’s Disease Rating Scale; WAIS, Weschler Adult Intelligence Test.

### Risk of bias

**Table 2** provides a summary of overall quality ratings. Three studies were rated as ‘good’ (56, 62, 63) and the remaining one (64) as ‘fair.’

**Table 2:** Quality assessment.

Study (Year)	Poor	Fair	Good
Aksu (62)			√
Manenti et al. (63)			√
Salisbury (64)		√	
Xu et al. (56)			√

**Figure 3:**
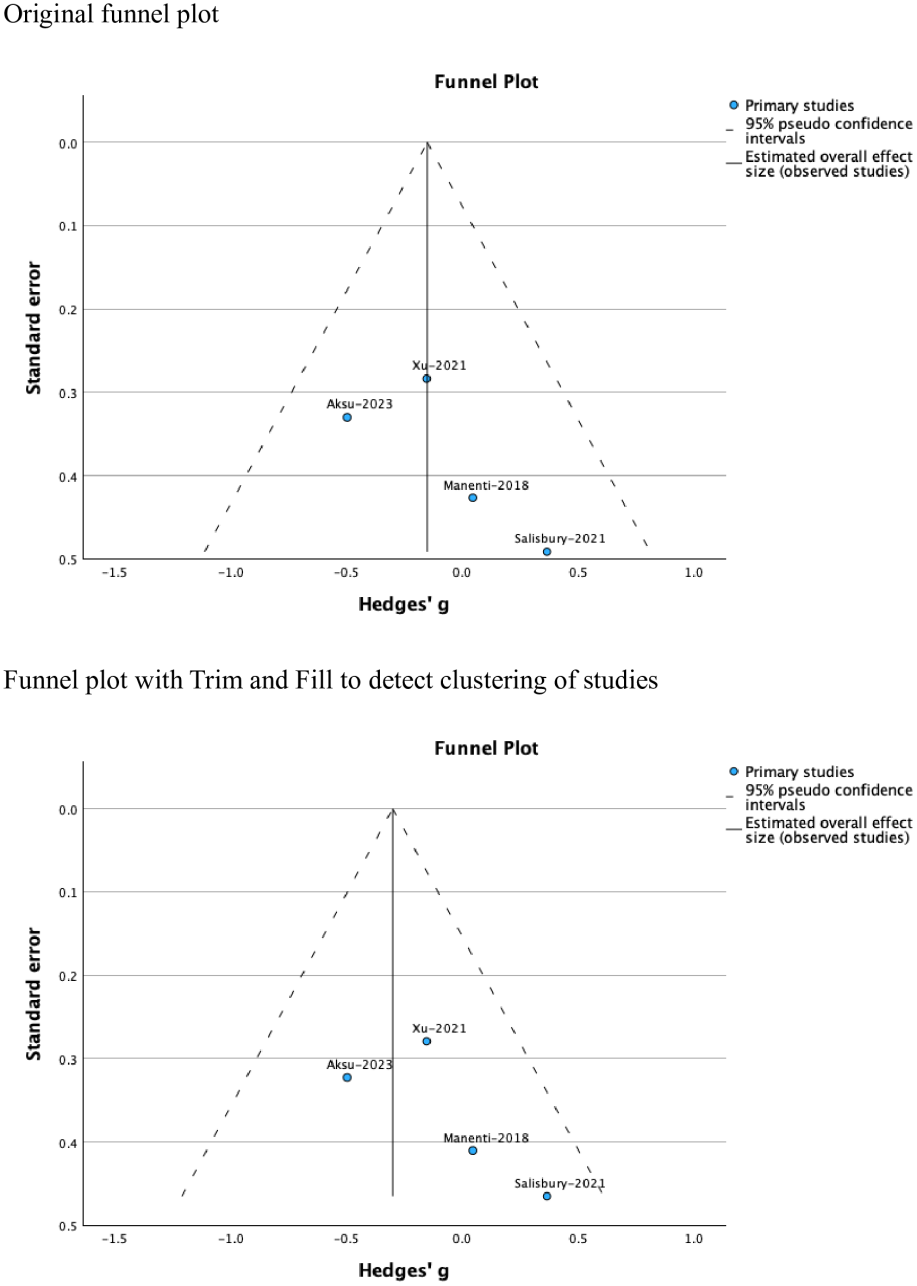
Funnel plots showing no evidence of publication bias.

Tests of heterogeneity were insignificant (Tau^2^ = 0.00, *H^2^ =* 1.00, *I^2^ =* 0.00). the funnel plot showed no evidence of publication bias (**Figure 3**). However, clinical heterogeneity related to the inclusion of individuals with different neuropsychic disorders and those with obesity was evident across the studies.

### Key findings

Given that facets of impulsivity correlate weakly with each other and have distinct neurobiological underpinnings (4), we elected to separately examine the effects of combined cognitive training and tDCS on different facets of impulsivity. Two studies measured motor impulsivity using the Stop Signal task (62) and a Go/No Go task (63). The statistical results are presented below under the relevant headings. It is worth noting that beyond statistics, we did not observe any specific trends across the different studies in relation to impulsivity outcomes.

### Motor impulsivity

Meta-analysis of the overall effects of combined NIBS and cognitive training on motor impulsivity or inhibitory control showed that the effects were insignificant (g= -0.15, 95% CT, -0.50 to 0.20, p=0.40) See **Figure 1**.

**Figure 4:**
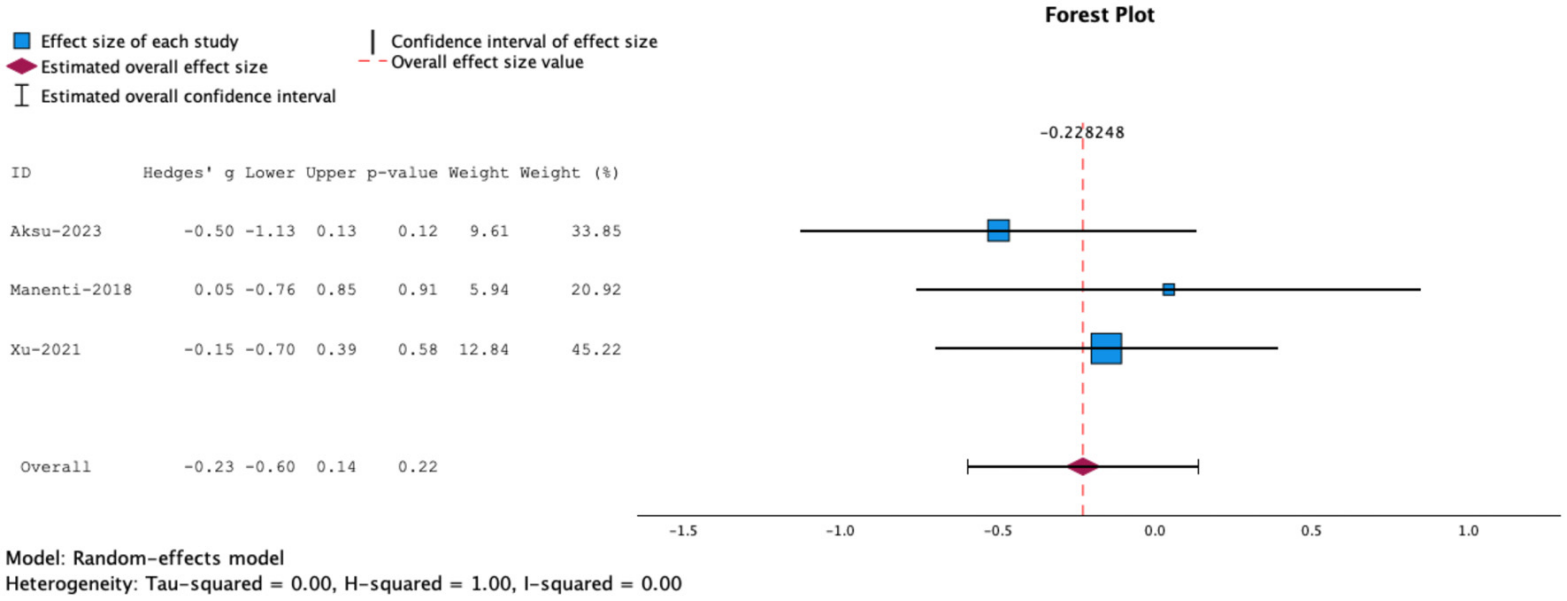
Forrest plot representing meta-analysis of Stop Signal Tasks. The overall effect size is not statistically significant.

Since the Flanker Task measures both attention and inhibitory control, we conducted a separate meta-analysis including SST and Go/No Go tasks. The results showed no statistically significant effects for combined NIBS and cognitive training on task performance (g= -0.23, 95% CT, -0.60 to 0.14, p=0.22) (**Figure 4**).

### Temporal impulsivity

One study reported pre and post stimulation data on delay discounting (56). The results showed no statistically significant difference in discounting scores between combined active tDCS and cognitive training and combined sham tDCS and cognitive training groups.

### Reflection impulsivity

None of the studies included in this review examined the effects of combined NIBS and cognitive training on reflection of cognitive impulsivity, highlighting the need for conducting studies of this kind.

## Discussion

In recent years, research interest has grown in combining tDCS with cognitive training paradigms to maximize training benefits, leading to task-specific cognitive enhancement. This review sought to systematically review the literature on the use of combined cognitive training and NIBS techniques to module impulsivity in adult populations. The results showed combined cognitive training and tDCS had no statistically significant effects on motor impulsivity (or inhibitory control) in people with substance use disorder, Parkinson’s disease, or obesity. While earlier meta-analyses of cognitive training research revealed mixed evidence for training effects and generalizability of effects to other tasks (65), more recent evidence suggests that combined tDCS and cognitive training can enhance performance on tasks designed to assess decision making (66) and inhibitory control (67). The findings this review adds to the uncertainty in this area.

Our findings may be related to the methodological limitations and clinical heterogeneity of the studies included in the review. For instance, it is notable that the cognitive training tasks used in this review were primarily related to cognitive functions like attention, processing speed, and working memory rather than impulsivity. It is likely that the enhancement effects of these tasks did not generalize to non-related or loosely related tasks that were designed to measure impulsivity. This is supported by previous evidence which shows that little or no evidence for transfer of training effects for inhibitory control (65, 68). It is also possible that the training tasks were inadequate in form or magnitude, highlighting the need to use impulsivity related training tasks, such as the Stop Signal Task, in future research. The utility of this task in improving inhibitory control was demonstrated in a study by Berkman et al. (67).

Furthermore, it is notable that the studies included in this review targeted the dorsolateral prefrontal cortex, although several neuronal circuits in the brain have been implicated in impulse control. These include the right inferior frontal gyrus, the anterior cingulated cortex, the basal ganglia, and the presupplementary motor area (5, 6), the ventromedial prefrontal cortex (7, 8) and the nucleus accumbens of the ventral striatum (9). Some of these structures are subcortical and may only be optimally stimulated through techniques like Deep TMS.

Achieving optimal results requires a thorough understanding of the mechanism of action of combined cognitive training which merits further investigation in future research. Existing evidence suggest that stimulating a neural network with tDCS while it is engaged by a cognitive stimulation task may be conducive to better therapeutic effects than stimulating the same neuronal network while lacking cognitive stimuli (46). Indeed, there is a suggestion that tDCS may increase the strength of synaptic transmission across pathways that are stimulated by cognitive training, leading a synergistic positive effect on behavior (44–46). However, a recent study that examined the mechanism of action of combined cognitive training and tDCS in adolescents with attention deficit hyperactivity disorder show no significant group differences in EEG spectral power on resting and Go/No Go task-based EEC measures (69).

Looking at the broader literature, there is evidence tDCS stimulation alone can reduce impulsivity in people with mental disorders (e.g., 32) and that concurrent cognitive training can enhance the neuroplasticity-related effects of tDCS (41) and improve decision making in clinically impulsive populations (40). There is also a suggestion that tDCS stimulation may reduce, rather than enhance, the effects of concurrent cognitive training through homeostatic down-regulation of brain networks (43). However, this is not supported by research showing that tDCS can enhance task related neuroplasticity (70) or a recent review which reported small but statistically significant effects for combined cognitive training and tDCS on attention, working memory, language and global cognitions in people with neuropsychiatric disorders (42). Furthermore, there is also a suggestion that the effects of tDCS can be enhanced by increasing the excitability of right dorsolateral prefrontal cortex (DLPFC) and reducing that of the left DLPFC through right anodal and left cathodal stimulation (56, 71). However, these effects were not demonstrated in the Xu et al. (56) study which involved a sample of individuals with methamphetamine use disorder.

The findings related to the use of combined cognitive training and tDCS to reduce inhibitory control in people with obesity are noteworthy (64). Research in this area yielded mixed results and our results add to this uncertainty. There is some preliminary evidence to support the use of combined cognitive training and tDCS to reduce caloric intake and enhance executive function in people with obesity (72). However, a small-scale study, that examined the effects of combined tDCS stimulation and the food choice task (FCT) on modifying food choice, craving, and consumption as a function of trait impulsivity found no differences in calorie intake between the active and sham groups (73). Ultimately, the etiology of obesity is multifactorial including genetic, psychosocial, and environmental factors, and the link between impulsivity and obesity is not fully understood. While research evidence suggests that impulsivity may play a role in development and maintenance of obesity in some individuals (55, 74), more research is needed to elucidate the role of impulsivity and whether this could be changed with combined neuromodulation and cognitive training.

### Strengths and limitations

A major strength of this study is its reliance on meta-analytic techniques to examine the effects of combined cognitive training and non-invasive brain stimulation on impulsivity and its subdomains. At the same time, the review was limited by the relatively small number of studies included in the review, which mainly involved males with substance use disorders. Notably, studies included in this study mostly focused on motor impulsivity and delay discounting and no studies measured reflection or cognitive impulsivity. Additionally, no study included participants based on impulsivity or used impulsivity as a primary outcome measure. These in conjunction with clinical heterogeneity limit the generalizability of findings to other populations such as those with other forms of major mental disorder and overall conclusions about impulsivity subdomains. The low sample size (n = 127 across four studies) additionally limits the statistical power and interpretation of the meta-analysis. Effect sizes may be under reported, and any biases in the included studies may be amplified. Finally, the search strategy included Scopus, PsychInfo, Medline, and CINHAL electronic databases in accordance with a recent review on combined cognitive training and tDCS (42). This may have missed studies listed in other relevant databases such as EMBASE.

### Future directions

Nascent literature in the field shows that tDCS and TMS, either as standalone interventions or combined with neurorehabilitation therapies, may positively alter neuroplasticity and improve neuropsychological, neuropsychiatric, motor, or somatic symptoms through brain stem activation (75). This mechanism, also known as the hormesis principle of neuroplasticity, has been proposed for explaining the use of NIBS to improve maladaptive brain physiology and behavioral symptoms resulting from acquired brain injury (76). Moreover, a recent imaging study identified reduced complexity in vmPFC as a putative mechanism for impulsivity in terms of risky and impatient economic choices (77). These putative mechanisms merit further investigations in studies involving the use of combined cognitive training and NIBS to modulate impulsivity and its subdomains. Future research should focus on conducting well designed studies involving disorders that are marked by impulsivity and including mechanistic evaluations such as brain imaging techniques, electroencephalography, and neurocognitive measures.

## Conclusion

There is a dearth of studies on the use of combined NIBS and cognitive training to modulate impulsivity and its subdomains. Studies included in this review only included participants with substance disorder, Parkinson’s disease and obesity. This limits the generalizability of the findings to other conditions that are marked by impulsivity such as attention deficit hyperactivity disorder, antisocial personality disorder and others. While the findings of this review show that concurrent NIBS and cognitive training has no statistically significant effects on motor impulsivity, the limitations inherent in the current literature preclude drawing definitive conclusions in this area. More research is required to advance knowledge in this field. Future studies should consider using training tasks that are designed to improve overall impulsivity and its subdomains. Future research should focus on conducting well designed studies in clinical populations with disorders that are marked by impulsivity such as those with ADHD, antisocial personality disorder, or borderline personality disorder. Future studies should include subdomains of impulsivity as primary outcomes measures, and include mechanistic evaluations such as brain imaging techniques, electroencephalography, and neurocognitive measures.

## Data Availability

The original contributions presented in the study are included in the article/**Supplementary Material**. Further inquiries can be directed to the corresponding author.

## References

[1] Castellanos-Ryan N Séguin JR . “Prefrontal and anterior cingulate cortex mechanisms of impulsivity.” In: Beauchaine TP Hinshaw SP , editors. The oxford handbook of externalizing spectrum disorders. Oxford University Press, New York (2015). p. 201–19. doi: 10.1093/oxfordhb/9780199324675.013.13

[2] Baker C Fairclough S Ogden RS Barnes R Tootill J . Trait impulsivity influences behavioural and physiological responses to threat in a virtual environment. Sci Rep. (2024) 14:9484. doi: 10.1038/s41598-024-60300-6 38664505 PMC11045749

[3] Cyders MA Smith GT . Mood-based rash action and its components: Positive and negative urgency. Pers. Indiv. Dif. (2007) 43:839–50. doi: 10.1016/j.paid.2007.02.008

[4] Yang CC Völlm B Khalifa N . The effects of rTMS on impulsivity in adults: a systematic review and meta-analysis. Neuropsychol. Rev. (2018) 28:377–92. doi: 10.1007/s11065-018-9376-6 29730846

[5] Wilbertz T Deserno L Horstmann A Neumann J Villringer A Heinze HJ . Response inhibition and its relation to multidimensional impulsivity. NeuroImage. (2014) 103:241–8. doi: 10.1016/j.neuroimage.2014.09.021 25241087

[6] Aron AR Fletcher PC Bullmore ET Sahakian BJ Robbins TW . 'Stop-signal inhibition disrupted by damage to right inferior frontal gyrus in humans’. Nat Neurosci. (2003) 6:115–6. doi: 10.1038/nn1003 12536210

[7] Bechara A Damasio AR Damasio H Anderson SW . Insensitivity to future consequences following damage to human prefrontal cortex. Cognition. (1994) 50:7–15. doi: 10.1016/0010-0277(94)90018-3 8039375

[8] Bechara A . The role of emotion in decision-making: evidence from neurological patients with orbitofrontal damage. Brain Cogn. (2004) 55:30–40. doi: 10.1016/j.bandc.2003.04.001 15134841

[9] Grant JE Kim SW . Brain circuitry of compulsivity and impulsivity. CNS Spectr. (2014) 19:21–7. doi: 10.1017/S109285291300028X 23659364

[10] Mitchell AS . The mediodorsal thalamus as a higher order thalamic relay nucleus important for learning and decision-making. Neurosci Biobehav Rev. (2015) 54:76–88. doi: 10.1016/j.neubiorev.2015.03.001 25757689

[11] Patton JH Stanford MS Barratt ES . Barratt impulsiveness scale-11 (BIS-11) [Database record. APA PsycTests. (1995). doi: 10.1037/t05661-000 8778124

[12] Logan GD . “On the ability to inhibit thought and action. A users’ guide to the stop signal paradigm.“. In: Dagenbach D Carr TH , editors. In Inhibitory processes in attention, memory and language. Academic Press, San Diego (1994). p. 189–236.

[13] Du W Green L Myerson J . Cross-cultural comparisons of discounting delayed and probabilistic rewards. Psychol Rec. (2002) 52:479–92. doi: 10.1007/BF03395199

[14] Gomez P Ratcliff R Perea M . A model of the go/no-go task. J Exp Psychol Gen. (2007) 136:389–413. doi: 10.1037/0096-3445.136.3.389 17696690 PMC2701630

[15] Clark L Robbins TW Ersche KD Sahakian BJ . Reflection impulsivity in current and former substance users. Biol Psychiatry. (2006) 60:515–22. doi: 10.1016/j.biopsych.2005.11.007 16448627

[16] American Psychiatric Association . Diagnostic and statistical manual of mental disorders: DSM-5. Arlington, VA: American Psychiatric Association (2013).

[17] Moore FR Doughty H Neumann T McClelland H Allott C O'Connor RC . Impulsivity, aggression, and suicidality relationship in adults: A systematic review and meta-analysis. EClinicalMedicine. (2022) 45:101307. doi: 10.1016/j.eclinm.2022.101307 35243273 PMC8860929

[18] Potvin S Pelletier J Grot S Hébert C Barr AM Lecomte T . Cognitive deficits in individuals with methamphetamine use disorder: A meta-analysis. Addict. Behav. (2018) 80:154–60. doi: 10.1016/j.addbeh.2018.01.021 29407687

[19] Wang YG Liu MH Shen ZH . A virtual reality counterconditioning procedure to reduce methamphetamine cue-induced craving. J Psychiatr Res. (2019) 116:88–94. doi: 10.1016/j.jpsychires.2019.06.007 31226580

[20] Moeller FG Dougherty DM Barratt ES Schmitz JM Swann AC Grabowski J . The impact of impulsivity on cocaine use and retention in treatment. J Subst Abuse Treat. (2001) 21:193–8. doi: 10.1016/s0740-5472(01)00202-1 11777668

[21] Anderson AC Youssef GJ Robinson AH Lubman DI Verdejo-Garcia A . Cognitive boosting interventions for impulsivity in addiction: a systematic review and meta-analysis of cognitive training, remediation and pharmacological enhancement. Addict. (Abingdon England). (2021) 116:3304–19. doi: 10.1111/add.15469 33751683

[22] Davis JP Barr N Dworkin ER Dumas TM Berey B DiGuiseppi G . Effect of mindfulness-based relapse prevention on impulsivity trajectories among young adults in residential substance use disorder treatment. Mindfulness. (2019) 10:1997–2009. doi: 10.1007/s12671-019-01164-0 32595783 PMC7318900

[23] Peckham AD Johnson SL . Cognitive control training for emotion-related impulsivity. Behav Res Ther. (2018) 105:17–26. doi: 10.1016/j.brat.2018.03.009 29609103 PMC5937944

[24] Tahir T Wong MM Maaz M Naufal R Tahir R Naidoo Y . Pharmacotherapy of impulse control disorders: A systematic review. Psychiatry Res. (2022) 311:114499. doi: 10.1016/j.psychres.2022.114499 35305343

[25] Van den Eynde F Senturk V Naudts K Vogels C Bernagie K Thas O . Efficacy of quetiapine for impulsivity and affective symptoms in borderline personality disorder. J Clin Psychopharmacol. (2008) 28:147–55. doi: 10.1097/JCP.0b013e318166c4bf 18344724

[26] Fregni F El-Hagrassy MM Pacheco-Barrios K Carvalho S Leite J Simis M . Evidence-based guidelines and secondary meta-analysis for the use of transcranial direct current stimulation in neurological and psychiatric disorders. Inter. J Neuropsychopharmacol. (2021) 24:256–313. doi: 10.1093/ijnp/pyaa051 PMC8059493 32710772

[27] Ozturk H Venugopal S . Transcranial magnetic stimulation as a therapeutic option for neurologic diseases and psychiatric disorders: A systematic review. Cureus. (2022) 14:e28259. doi: 10.7759/cureus.28259 36158376 PMC9491149

[28] Sullivan CRP Olsen S Widge AS . Deep brain stimulation for psychiatric disorders: From focal brain targets to cognitive networks. NeuroImage. (2021) 225:117515. doi: 10.1016/j.neuroimage.2020.117515 33137473 PMC7802517

[29] Martinotti G Lupi M Montemitro C Miuli A Di Natale C Spano MC . Transcranial direct current stimulation reduces craving in substance use disorders: A double-blind, placebo-controlled study. J ECT. (2019) 35:207–11. doi: 10.1097/YCT.0000000000000580 30844881

[30] Mahoney JJ Hanlon CA Marshalek PJ Rezai AR Krinke L . Transcranial magnetic stimulation, deep brain stimulation, and other forms of neuromodulation for substance use disorders: Review of modalities and implications for treatment. J Neurol Sci. (2020) 418:117149. doi: 10.1016/j.jns.2020.117149 33002757 PMC7702181

[31] Teti Mayer J Chopard G Nicolier M Gabriel D Masse C Giustiniani J . Can transcranial direct current stimulation (tDCS) improve impulsivity in healthy and psychiatric adult populations? A systematic review. Prog Neuro-psychopharmacol. Biol Psychiatry. (2020) 98:109814. doi: 10.1016/j.pnpbp.2019.109814 31715284

[32] Yang CC Mauer L Völlm B Khalifa N . The effects of non-invasive brain stimulation on impulsivity in people with mental disorders: A systematic review and explanatory meta-analysis. Neuropsychol. Rev. (2020) 30:499–520. doi: 10.1007/s11065-020-09456-2 33009976

[33] Nitsche MA Müller-Dahlhaus F Paulus W Ziemann U . The pharmacology of neuroplasticity induced by non-invasive brain stimulation: building models for the clinical use of CNS active drugs. J Physiol. (2012) 590:4641–62. doi: 10.1113/jphysiol.2012.232975 PMC3487028 22869014

[34] Polanía R Nitsche MA Ruff CC . Studying and modifying brain function with non-invasive brain stimulation. Nat Neurosci. (2018) 21:174–87. doi: 10.1038/s41593-017-0054-4 29311747

[35] Gobet F Sala G . Cognitive training: A field in search of a phenomenon. Perspect Psychol Sci. (2023) 18:125–41. doi: 10.1177/17456916221091830 PMC9903001 35939827

[36] Sala G Aksayli ND Tatlidil KS Tatsumi T Gondo Y Gobet F . Near and far transfer in cognitive training: A second-order meta-analysis. Collabra. Psychol. (2019) 5:18. doi: 10.1525/collabra.203

[37] Sampedro-Piquero P Ladrón de Guevara-Miranda D Pavón FJ Serrano A Suárez J Rodríguez de Fonseca F . Neuroplastic and cognitive impairment in substance use disorders: a therapeutic potential of cognitive stimulation. Neurosci Biobehav Rev. (2019) 106:23–48. doi: 10.1016/j.neubiorev.2018.11.015 30481530

[38] Leung IH Walton CC Hallock H Lewis SJ Valenzuela M Lampit A . Cognitive training in Parkinson disease: A systematic review and meta-analysis. Neurology. (2015) 85:1843–51. doi: 10.1212/WNL.0000000000002145 PMC4662707 26519540

[39] Díez-Cirarda M Ojeda N Peña J Cabrera-Zubizarreta A Lucas-Jiménez O Gómez-Esteban JC . Long-term effects of cognitive rehabilitation on brain, functional outcome and cognition in Parkinson's disease. Eur J Neurol. (2018) 25:5–12. doi: 10.1111/ene.13472 28940855 PMC5765471

[40] Gilmore CS Dickmann PJ Nelson BG Lamberty GJ Lim KO . Transcranial Direct Current Stimulation (tDCS) paired with a decision-making task reduces risk-taking in a clinically impulsive sample. Brain Stim. (2018) 11:302–9. doi: 10.1016/j.brs.2017.11.011 29174303

[41] Bikson M Name A Rahman A . Origins of specificity during tDCS: anatomical, activity-selective, and input-bias mechanisms. Front Hum Neurosci. (2013) 7:688. doi: 10.3389/fnhum.2013.00688 24155708 PMC3800813

[42] Burton CZ Garnett EO Capellari E Chang SE Tso IF Hampstead BM . Combined cognitive training and transcranial direct current stimulation in neuropsychiatric disorders: A systematic review and meta-analysis. Biol Psychiatry Cogn. Neurosci Neuroimaging. (2023) 8:151–61. doi: 10.1016/j.bpsc.2022.09.014 PMC10823589 36653210

[43] Elmasry J Loo C Martin D . A systematic review of transcranial electrical stimulation combined with cognitive training. Restor. Neurol Neurosci. (2015) 33:263–78. doi: 10.3233/RNN-140473 25624425

[44] Miniussi C Harris JA Ruzzoli M . Modelling non-invasive brain stimulation in cognitive neuroscience. Neurosci Biobehav Rev. (2013) 37:1702–12. doi: 10.1016/j.neubiorev.2013.06.014 23827785

[45] Birba A Ibáñez A Sedeño L Ferrari J García AM Zimerman M . Non-invasive brain stimulation: A new strategy in mild cognitive impairment? Front Aging Neurosci. (2017) 13:16. doi: 10.3389/fnagi.2017.00016 PMC5303733 28243198

[46] Cruz Gonzalez P Fong KNK Brown T . The effects of transcranial direct current stimulation on the cognitive functions in older adults with mild cognitive impairment: A pilot study. Behav Neurol. (2018) 15:5971385. doi: 10.1155/2018/5971385 PMC5874987 29736192

[47] Falissard B Coghill D Rothenberger A Lorenzo M ADORE Study Group . Short-term effectiveness of medication and psychosocial intervention in a cohort of newly diagnosed patients with inattention, impulsivity, and hyperactivity problems. J Atten Disord. (2010) 14:147–56. doi: 10.1177/1087054709347173 19767593

[48] Tourjman V Louis-Nascan G Ahmed G DuBow A Côté H Daly N . Psychosocial interventions for attention deficit/hyperactivity disorder: A systematic review and meta-analysis by the CADDRA guidelines work GROUP. Brain Sci. (2022) 12:1023. doi: 10.3390/brainsci12081023 36009086 PMC9406006

[49] Mechler K Banaschewski T Hohmann S Häge A . Evidence-based pharmacological treatment options for ADHD in children and adolescents. Pharmacol Ther. (2022) 230:107940. doi: 10.1016/j.pharmthera.2021.107940 34174276

[50] Moher D Liberati A Tetzlaff J Altman DG PRISMA Group . Preferred reporting items for systematic reviews and meta-analyses: the PRISMA statement. PloS Med. (2009) 6:e1000097. doi: 10.1371/journal.pmed.1000097 19621072 PMC2707599

[51] Clare L Woods RT . Cognitive training and cognitive rehabilitation for people with early-stage Alzheimer's disease: A review, Neuropsychol. Rehabil. (2004) 14:385–401. doi: 10.1080/09602010443000074

[52] Verdejo-Garcia A Lawrence AJ Clark L . Impulsivity as a vulnerability marker for substance-use disorders: review of findings from high-risk research, problem gamblers and genetic association studies. Neurosci Biobehav Rev. (2008) 32:777–810. doi: 10.1016/j.neubiorev.2007.11.003 18295884

[53] Caswell AJ Bond R Duka T Morgan MJ . Further evidence of the heterogeneous nature of impulsivity. Pers. Indiv. Dif. (2015) 76:68–74. doi: 10.1016/j.paid.2014.11.059 PMC4316178 25844002

[54] Riley RD Higgins JP Deeks JJ . Interpretation of random effects meta-analyses. BMJ (Clinical Res ed.). (2011) 342:d549. doi: 10.1136/bmj.d549 21310794

[55] Carters MA Rieger E Bell J . Reduced inhibition of return to food images in obese individuals. PloS One. (2015) 10:e0137821. doi: 10.1371/journal.pone.0137821 26376082 PMC4574472

[56] Xu X Ding X Chen L Chen T Su H Li X . The transcranial direct current stimulation over prefrontal cortex combined with the cognitive training reduced the cue-induced craving in female individuals with methamphetamine use disorder: A randomized controlled trial. J Psychiatr Res. (2021) 134:102–10. doi: 10.1016/j.jpsychires.2020.12.056 33383492

[57] Higgins JP Thompson SG . Quantifying heterogeneity in a meta-analysis. Stat Med. (2002) 21:1539–58. doi: 10.1002/sim.1186 12111919

[58] Cochrane Collaboration . Cochrane handbook: general methods for cochrane reviews (2014). Available online at: https://handbook-5-1.cochrane.org/chapter_9/9_5_heterogeneity.htm (Accessed 25 August 2024).

[59] Littell JH Corcoran J Pillai V . *Systematic reviews and meta-analysis*, pocket guides to social work research methods (2008). New York: Oxford Academic. Available online at: 10.1093/acprof:oso/9780195326543.001.0001 (Accessed 19 July 2024).

[60] Egger M Davey Smith G Schneider M Minder C . Bias in meta-analysis detected by a simple, graphical test. BMJ. (1997) 315:629–34. doi: 10.1136/bmj.315.7109.629 PMC2127453 9310563

[61] NIH National Heart, Lung, and Blood Institute . Quality assessment tool for observational, cohort and cross-sectional studies . Available online at: https://www.nhlbi.nih.gov/health-pro/guidelines/in-develop/cardiovascular-risk-reduction/tools/cohort (Accessed September 25, 2024).

[62] Aksu S Soyata AZ Şeker S Akkaya G Yılmaz Y Kafalı T . Transcranial direct current stimulation combined with cognitive training improves decision making and executive functions in opioid use disorder: a triple-blind sham-controlled pilot study. J.Addict. Dis. (2024) 42:154–65. doi: 10.1080/10550887.2023.2168991 36861945

[63] Manenti R Cotelli MS Cobelli C Gobbi E Brambilla M Rusich D . Transcranial direct current stimulation combined with cognitive training for the treatment of Parkinson Disease: A randomized, placebo-controlled study. Brain Stim. (2018) 11:1251–62. doi: 10.1016/j.brs.2018.07.046 30056141

[64] Salisbury M . The Role of Transcranial Direct Current Stimulation and Cognitive Training to Decrease Food-Related Impulsivity Behavior in Individuals with Obesity: a Review and Pilot Study. [Dissertations/master’s thesis]. [University Digital Conservancy]. Minnesota, United States, University of Minnesota (2021). Available at: https://hdl.handle.net/11299/224456.

[65] Owen AM Hampshire A Grahn JA Stenton R Dajani S Burns AS . Putting brain training to the test. Nature. (2010) 465:775–8. doi: 10.1038/nature09042 PMC2884087 20407435

[66] Filmer HL Varghese E Hawkins GE Mattingley JB Dux PE . Improvements in attention and decision-making following combined behavioral training and brain stimulation. Cerebr. Cortex. (2017) 27:3675–82. doi: 10.1093/cercor/bhw189 27436130

[67] Berkman ET Kahn LE Merchant JS . Training-induced changes in inhibitory control network activity. J Neurosci. (2014) 34:149–57. doi: 10.1523/JNEUROSCI.3564-13.2014 PMC3866481 24381276

[68] Melby-Lervåg M Hulme C . Is working memory training effective? A meta-analytic review. Dev Psychol. (2013) 49:270–91. doi: 10.1037/a0028228 22612437

[69] Westwood SJ Bozhilova N Criaud M Lam SL Lukito S Wallace-Hanlon S . The effect of transcranial direct current stimulation (tDCS) combined with cognitive training on EEG spectral power in adolescent boys with ADHD: A double-blind, randomized, sham-controlled trial. IBRO Neurosci Rep. (2021) 12:55–64. doi: 10.1016/j.ibneur.2021.12.005 35746969 PMC9210460

[70] Pisoni A Mattavelli G Papagno C Rosanova M Casali AG Romero Lauro LJ . Cognitive enhancement induced by anodal tDCS drives circuit-specific cortical plasticity. Cereb Cortex. (2018) 28:1132–40. doi: 10.1093/cercor/bhx021 28184424

[71] Lefaucheur JP Antal A Ayache SS Benninger DH Brunelin J Cogiamanian F . Evidence-based guidelines on the therapeutic use of transcranial direct current stimulation (tDCS). Clin Neurophysiol. (2017) 128:56–92. doi: 10.1016/j.clinph.2016.10.087 27866120

[72] Forcano L Castellano M Cuenca-Royo A Goday-Arno A Pastor A Langohr K . Prefrontal cortex neuromodulation enhances frontal asymmetry and reduces caloric intake in patients with morbid obesity. Obesity. (2020) 28:696–705. doi: 10.1002/oby.22745 32144883

[73] Georgii C Goldhofer P Meule A Richard A Blechert J . Food craving, food choice and consumption: The role of impulsivity and sham-controlled tDCS stimulation of the right dlPFC. Physiol Behav. (2017) 177:20–6. doi: 10.1016/j.physbeh.2017.04.004 28396289

[74] Bartholdy S Dalton B O'Daly OG Campbell IC Schmidt U . A systematic review of the relationship between eating, weight and inhibitory control using the stop signal task. Neurosci Biobehav Rev. (2016) 64:35–62. doi: 10.1016/j.neubiorev.2016.02.010 26900651

[75] Mattson MP Leak RK . The hormesis principle of neuroplasticity and neuroprotection. Cell Metab. (2024) 36:315–37. doi: 10.1016/j.cmet.2023.12.022 38211591

[76] Eliason M Kalbande PP Saleem GT . Is non-invasive neuromodulation a viable technique to improve neuroplasticity in individuals with acquired brain injury? A review. Front Hum Neurosci. (2024) 18:1341707. doi: 10.3389/fnhum.2024.1341707 39296918 PMC11408216

[77] Bergström F Lerman C Kable JW . Less cortical complexity in ventromedial prefrontal cortex is associated with a greater preference for risky and immediate rewards. bioRxiv: preprint server Biol. (2023) 09:12. doi: 10.1101/2023.09.12.557368 PMC12247602 40800479

